# A large-scale survey on epidemiology and underreporting of needlestick and sharp injuries among healthcare workers in China

**DOI:** 10.3389/fpubh.2023.1292906

**Published:** 2023-11-02

**Authors:** Wang Tonghui, Liang Ying, Wu Xiaolu, Hao Ming

**Affiliations:** ^1^Department of Public Health, Lanzhou University Second Hospital, Lanzhou, China; ^2^Department of Medical Affairs, Lanzhou University Second Hospital, Lanzhou, China

**Keywords:** needlestick and sharp injuries, underreporting, training, health care workers (HCWs), China

## Abstract

**Background:**

Needlestick and sharp injuries (NSI) carry the risk of transmitting numerous bloodborne pathogens, leading to both health and economic burdens. The underreporting of NSIs among healthcare workers (HCWs) is a global issue of concern, as timely treatment and prevention of complications rely on proper reporting. Underreporting further impedes accurate surveillance and appropriate resource allocation, with developed and developing nations facing disparities due to differences in healthcare policy.

**Purpose:**

The purpose of this research is to examine the epidemiology of NSIs and NSI underreporting, as well as to identify the determinants associated with the occurrence of NSIs and the underreporting of such injuries.

**Method:**

A retrospective online survey was conducted from January 15 to January 31, 2022 among healthcare workers (HCWs) across Gansu Province, China.

**Results:**

A total of 7,283 healthcare workers (HCWs) from various institutions participated in this study. After quality assurance checks, 6,464 (88.77%) responses were included in the final analysis. Results revealed a 32.86% self-reported needlestick and sharp injury (NSI) incidence among respondents, with 28.53% of NSIs going unreported. Contrary to common belief, more experienced HCWs exhibited higher rates of both NSIs and underreporting compared to their less experienced peers. The primary reasons cited for NSIs and underreporting were lapses in concentration and not perceiving patients as infectious. Multivariate regression analysis exposes the significant influence of training frequency, occupation, department and years of services on the occurrence of NSIs. Conversely, the reporting of NSIs is primarily influenced by training, reimbursement,occupation, department and hospital grade. Compared to HCWs with no training, those who received ≥3 training sessions per year showed a 12.16% lower NSI incidence (27.12% vs. 39.28%, *p* < 0.001) and a 55.68% lower underreporting rate (14.61% vs. 70.29%, *p* < 0.001).

**Conclusion:**

There is a pressing need for enhanced surveillance, tailored training programs, and more efficient reporting mechanisms to combat this significant occupational health challenge.

## Background

Needlestick and sharp injuries (NSI) is one of the most prevalent occupational hazards encountered by healthcare workers (HCWs) (1). Other than hepatitis B virus (HBV), hepatitis C virus (HCV), and human immunodeficiency virus (HIV), these injuries can transmit over 20 dangerous bloodborne pathogens that may result in severe or even fatal health outcomes (2). Beyond the risk of bloodborne infections, NSIs confer significant psychological (3, 4) and economic burdens on affected HCWs (5). According to estimates by the World Health Organization (WHO), approximately 35 million NSIs occur among HCWs annually on a global scale (6). Some scholars estimate there could be as many as 3.8 million NSIs among HCWs in China annually, a figure ten times greater than estimates for the United States (2, 7).

NSI underreporting is relatively common, occurring due to concerns over the perceived severity of the injury, social bias, or Cumbersome reporting procedures (8). Behzadmehr et al.’s meta-analysis containing 41 studies highlights a positive correlation between NSIs reporting rates and socio-economic development. Notably, low-income countries exhibit a 75% underreporting rate, middle to high-income countries 61.5%, and high-income countries 52.4%. Geographically, Southeast Asia reports the highest at 87.9%, while the United States reports the lowest at 47.8% (6). This discrepancy can be attributed to the comparatively inadequate public health infrastructure and regulatory frameworks in these developing countries (6, 9, 10). Reporting is crucial to enable timely treatment and prevent complications. Underreporting impedes accurate monitoring and resource allocation, necessitating improved surveillance to address this global public health issue.

Occupational differences exist in both the prevalence and reporting of NSIs among HWCs. Studies have examined NSIs among various HWCs professions, including nursing students (8), dentists (11, 12), resident physicians (13–15), surgeons (16), and OR nurses (17). Significant variations are observed in the frequency and reporting habits between these groups (8, 12, 14, 18, 19). However, the epidemiology of underreported NSIs remains unclear, with limited multi-center studies and ambiguous evidence on influencing factors. Few studies have thoroughly investigated underreporting behaviors with large sample sizes. Often, when addressing NSI underreporting rates, there is a lack of in-depth exploration into crucial individual and organizational factors, such as shame, fear, training, and safety culture. Furthermore, determinants of NSI underreporting are not well defined. This research aims to help address these knowledge gaps through a comprehensive investigation of NSI epidemiology among thousands of HCWs in diverse hospital settings across Gansu Province, China. We hope to uncover factors impacting NSI prevalence and underreporting rates and the influence of focused training.

## Methods

### Study site

Gansu Province is located in northwest China, with a population of 25 million as of 2021 (20). Gansu ranked second last among all provinces in GDP *per capita* in 2019 (21). As one of the most underdeveloped provinces in the country, Gansu faces significant economic constraints and healthcare resource shortages. By the end of 2021, Gansu had only 2.84 practicing physicians per 1,000 population, lower than the national average of 3.04. The healthcare infrastructure includes 699 hospitals and 24,373 primary care clinics (20). However, accessibility and service quality remain challenging, especially in rural areas.

### Study population

As a large, underdeveloped province, Gansu’s healthcare delivery relies heavily on its county-level hospitals and township health centers network. We strived for a representative sample by including all major public hospitals and primary care sites from urban and rural areas. This study included 646 public hospitals across Gansu Province, comprising 361 general hospitals, 116 traditional Chinese medicine hospitals, and 169 specialized hospitals. Additionally, 698 community health centers were enrolled. However, health centers and clinics situated in cities below the county level, as well as health facilities without inpatient beds were excluded, given the infeasibility of gathering reliable statistics from such fragmented settings. Due to similar scope of practice and staff counts, community health centers and township hospitals were analyzed jointly. All HCWs were categorized into three occupational groups: physicians, nurses, and others (including technicians, anesthetists, laboratory staff, and janitorial staff). Ethics approval was obtained from the Lanzhou University Second Hospital review board with the waiver of informed consent. All methods were carried out in accordance with the Declaration of Helsinki and relevant guidelines and regulations.

### Data collection and definition

This study utilized an online questionnaire survey distributed to healthcare facilities through provincial and local health authorities. An online platform was chosen to efficiently reach the large number of HCWs across Gansu province. Between January 15 and 31, 2022, a survey was conducted, accessed through a QR code to an online platform. Notably, the survey focused on NSIs and reporting states, specifically those occurring between January 14, 2021, and January 15, 2022. It’s important to highlight that the questionnaire maintained anonymity, refraining from collecting personal identifiers like names or ID numbers to promote candid and unbiased responses.The questionnaire comprised 12 items, including seven demographic questions and five items on occupational exposures and associated factors (Supplementary File S1).

### Statistical analysis

The primary outcome in this study is the NSI reporting rate, while the secondary outcome is the overall NSI prevalence. The study started by conducting a descriptive analysis of the distribution of NSI prevalence and reporting rates across various demographic categories. A chi-square test was employed to ascertain the gap among distinct demographic groups.

Moreover, the investigation proceeds to validate the factors contributing to the occurrence and non-reporting of NSI among HCWs with varying years of service. This examined the reasons behind the occurrence and non-reporting of NSIs, differentiating across personnel with diverse lengths of service. Furthermore, by employing the Margin model, the study calculates the marginal impact of differing training frequencies and diverse reimbursement claims on the incidence and reporting rates of NSIs based on the results of logistical regressions. The study adopts a significance threshold of *p* < 0.05 to establish statistical significance. The study used Microsoft Office 2016 for data management and Stata 17 for comprehensive statistical computation and analysis.

## Results

A total of 7,283 HCWs from various institutions participated in the study. 6,464 (88.77%) of these responses passed the quality assurance checks. 2,134 (32.86%) reported experiencing NSIs. Among those who had experienced an NSI, 606 (28.53%) did not report the incident. The sample included 1,681 HCWs from primary hospitals, 2,683 from secondary hospitals, and 2,100 from tertiary hospitals. There were 4,873 females and 1,591 males.

Table 1 reports the characteristics of NSIs and underreporting among 6,464 HCWs across different healthcare institutions, departments, and demographic groups. By healthcare institution, tertiary hospitals had the highest NSI rate at 773 (36.81%) compared to 500 (29.74%) in primary hospitals (*p* < 0.001). The surgery department had the highest NSI rate at 360 (43.22%), while other departments had the lowest at 502 (26.08%) (*p* < 0.001). Regarding underreporting, emergency department staff had the lowest rate at 15 (16.13%) versus 274 (31.99%) from internal medicines (*p* < 0.001).

**Table 1:** Characteristics of NSIs and underreported NSIs by HCWs.

	*N* = 6,464	NSIs	%	***P***	Underreported	%	***P***
**Healthcare institution**				<0.001			<0.001
Primary hospital	1,681	500	29.74		248	49.60	
Secondary hospital	2,683	861	32.09		190	22.07	
Tertiary hospital	2,100	773	36.81		168	21.73	
**Gender**				0.382			<0.001
Femal	4,873	1,623	33.31		426	26.25	
Male	1,591	511	32.12		180	35.23	
**Department**				<0.001			0.006
Pediatric	464	165	35.56		48	29.09	
Other departments	1,925	502	26.08		151	30.08	
Internal medicines	2,432	772	31.74		247	31.99	
Surgery	833	360	43.22		88	24.44	
Obstetrics and gynecology	475	191	40.21		44	23.04	
Emergency department	205	93	45.37		15	16.13	
Intensive care unit	130	51	39.23		13	25.49	
**Education**				0.303			0.646
Junior college	2,742	880	32.09		259	29.43	
Undergraduate	3,659	1,230	33.62		341	27.72	
Graduate or above	63	24	38.10		6	25.00	
**Professional title**				<0.001			0.121
Ungraded	978	262	26.79		84	32.06	
Primary	3,457	1,132	32.75		297	26.24	
Medium-grade	1,464	520	35.52		159	30.58	
Senior	565	220	38.94		66	30.00	
**Years of service**				<0.001			0.010
≤1	559	144	25.76		31	21.53	
>1- ≤ 5	1,535	504	32.83		125	24.80	
>5	4,370	1,486	34.00		450	30.28	
**Occupation**				<0.001			<0.001
Other	694	142	20.46		113	79.54	
Doctor	2,544	790	31.05		545	68.95	
Nurse	3,226	1,065	33.01		668	62.74	

Overall, increased training frequency lowered NSI incidence and un-reporting rate (*p* < 0.001). Specifically, ≥3 training per year resulted in a 12.16% lower incidence (27.12% vs. 39.28%, *p* < 0.001) and a 55.68% lower underreporting rate (14.61% vs. 70.29% p < 0.001) versus no training. Full reimbursement of costs following occupational exposure was associated with a 29.37% higher reporting rate than unclear policies (83.09% vs. 53.72%, *p* < 0.001) (Table 2).

**Table 2:** Effect of training and reimburse on NSIs and report rate.

	*N* = 6,464	NSIs	%	***P***	*N* = 2,134	Underreported	%	***P***
**Training on occupational exposure prevention and management**				<0.001				<0.001
No	791	276	34.89		276	194	70.29	
1/year	3,060	1,008	32.94		1,008	271	26.88	
2/year	1,061	412	38.83		412	77	18.69	
≥3/year	1,552	438	28.22		438	64	14.61	
**Cost coverage following occupational exposure**								<0.001
Unclear					121	56	46.28	
Borne by the individual					875	357	40.80	
50% by the individual and 50% by the hospital					328	56	17.07	
Reimbursed by the employer					810	137	16.91	

**Figure 1:**
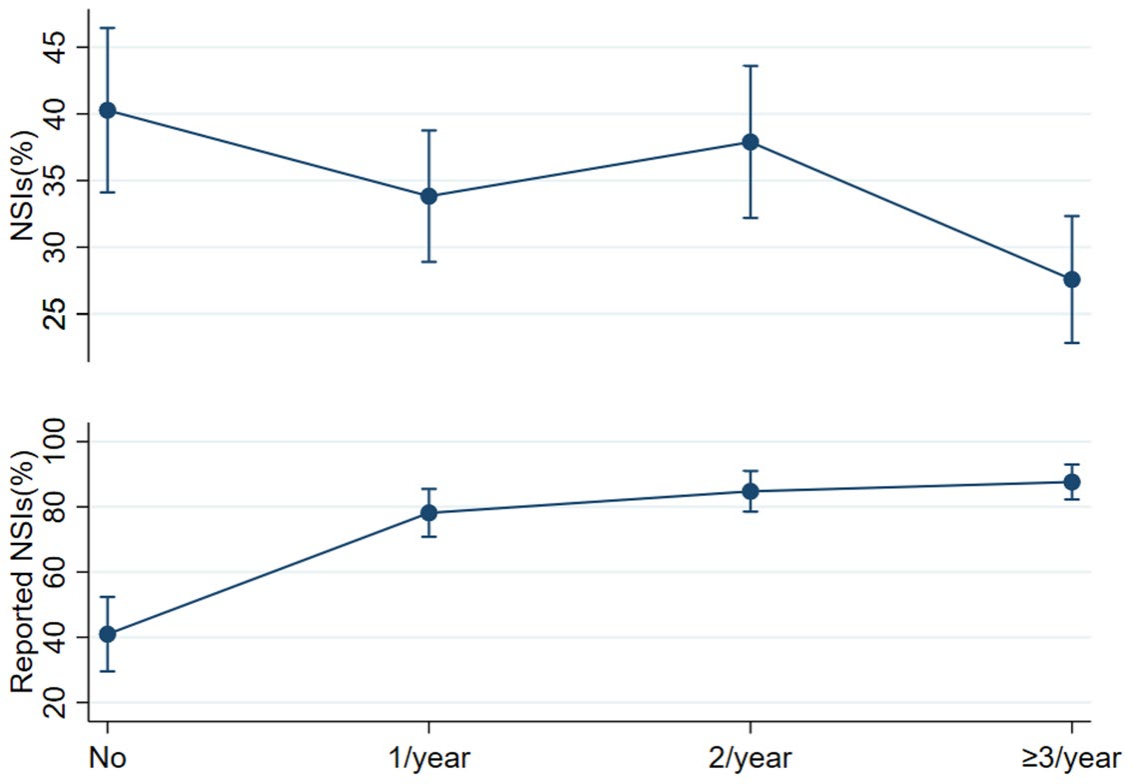
Estimate NSIs and report rate with varying training.

Figure 1 examines the marginal impact of training frequency on NSI prevalence and reporting rates. Increased training correlates with lower NSI prevalence and higher reporting rates. Specifically, compared to no training, ≥3 trainings per year reduces the predicted NSI probability by 12.15%. More frequent training also enhances reporting compliance, with the expected reporting probability rising by 30.54 to 39.76% relative to no training, depending on training frequency (Figure 1). This analysis is based on two logistic regression results derived from the supporting file: one encompassing all 6,464 survey participants, as HCWs with over 5 years of service exhibit elevated odds (OR 1.28), while nurses demonstrate a substantial increase in risk (OR 1.93). More frequent training sessions, especially ≥3/year, are associated with significantly lower odds of NSIs (OR 0.56)(Supporting file 2). Another focusing on the subset of 2,134 participants who experienced NSI. Reveal secondary hospitals (OR = 1.98) exhibit increased odds of underreporting, contrasting with doctors who display significantly lower odds (OR = 0.53). Higher training frequency, notably ≥3 sessions/year, substantially raises the odds of underreporting (OR 8.41)(Supporting file 3). Despite the diversity in NSI occurrences, our multivariate regression analysis did not reveal significant differences based on tenure for unreported NSI.

**Figure 2:**
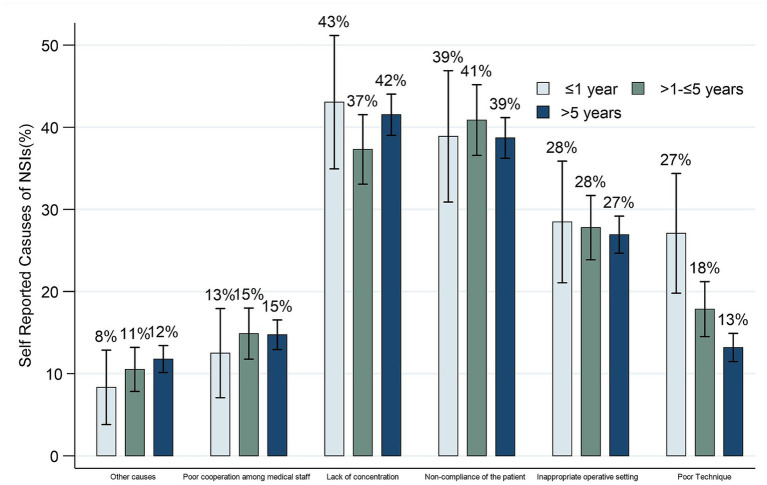
Self-reported causes of NSIs among HCWs with varying years of service.

NSIs can occur multiple times within a year, and various factors may precipitate each incident. Therefore, questions regarding the reasons for NSIs allowed multiple choices. Figure 2 examines self-reported causes of NSIs among HCWs with varying years of service. Lack of concentration was the most common contributor across all experience groups. Non-compliance of patients was similar across groups (39–41%). The <1-year group reported the highest rates of poor surgical/procedural technique (27%). Lack of concentration decreased slightly from 43% (<1-year) to 37% (1–5 years), then rose to 42% (>5-years). Poor cooperation among staff was consistent (13–15%).

**Figure 3:**
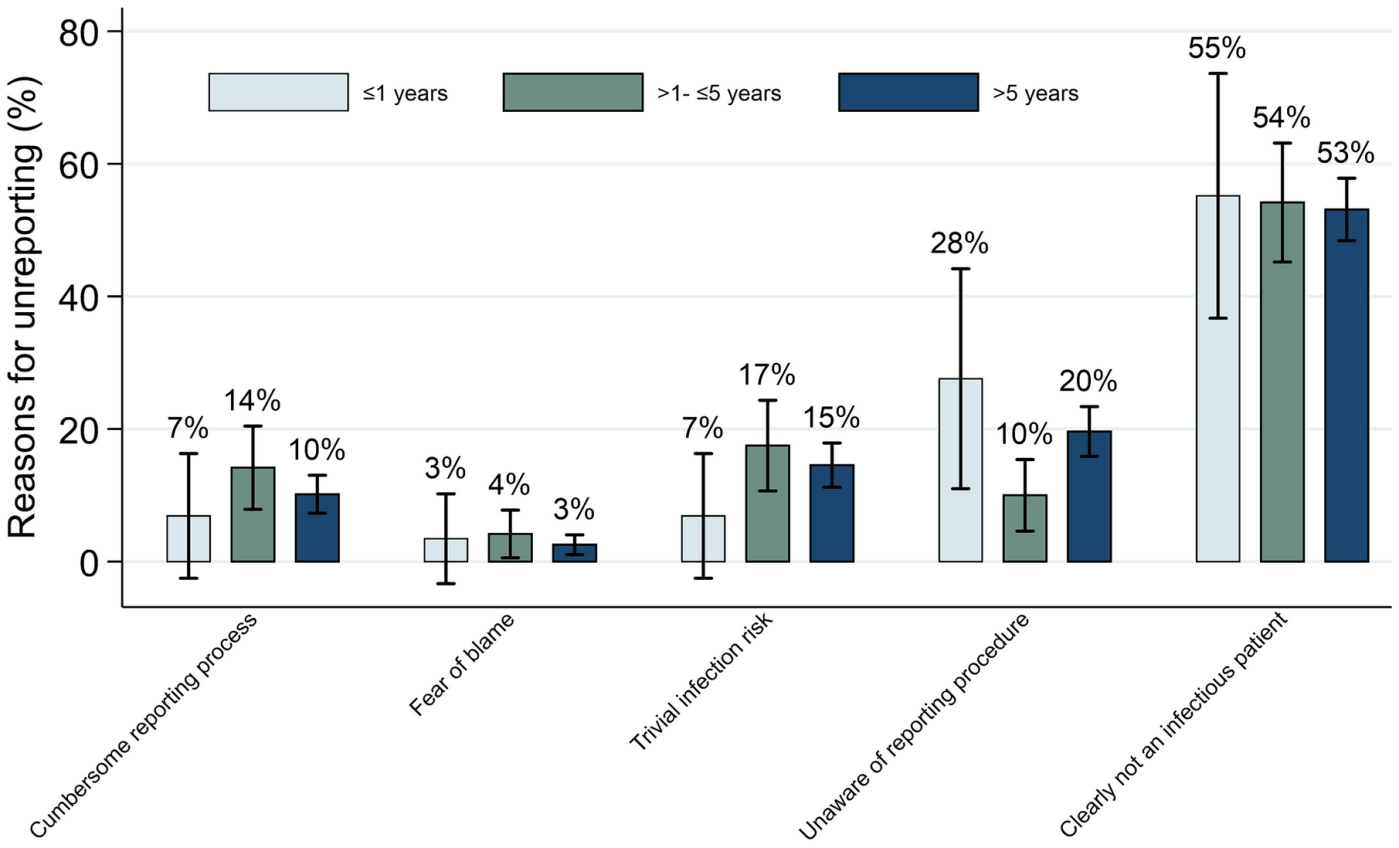
Self-reported reasons for not reporting among HCWs with varying years of service (*N* = 972).

Figure 3 examines self-reported reasons for not reporting NSIs among HCWs across varying years of service. Not perceiving the patient as infectious was the top reason for underreporting in all groups, though this decreased slightly from 55.8% (≤1-year) to 48.5% (>5-years). Unawareness of reporting procedures was higher in less experienced groups (26.9% for ≤1-year vs. 14.7% for 1–5 years). Perceiving trivial infection risk and cumbersome processes were also more significant concerns among experienced HCWs. Fear of blame remained low and consistent. Notable differences emerged in the perceived infectiousness of the patient and awareness of reporting protocols based on years of experience.

## Discussion

The self-reported 32.86% NSI prevalence in this study is notably higher than the rates observed in developed countries such as Australia (16.6%) (15) and Switzerland (9.7%) (22). Extensive research has demonstrated a strong correlation between NSI prevalence and socio-economic development, influenced by factors including workforce allocation, safety culture, and equipment availability (6, 9, 23). Hence, it is justifiable to suggest that the lower NSI rates found in studies conducted in underdeveloped regions with limited reporting systems could be due to reduced reporting. This perspective offers a plausible explanation for the elevated NSI rate in this study compared to the rates in Nigeria (24.5%) (24) and Thailand (23.7%) (17), both of which possess lower economic and social standing than China. Furthermore, the study findings also indicate that the NSI prevalence within Gansu Province is notably lower than that of several underdeveloped nations, such as Iran (81.7, 54%) (25, 26), and India (49.1, 35.3%) (27, 28), thereby illustrating regional variations within the broader context of global disparities. The research findings reveal a noteworthy underreporting rate of 28.53% for NSI, a figure significantly below the wide range of 95.4 to 39.7% reported by various scholars in surveys across different regions of China (3, 8, 29–33). This rate also remarkably contrasts with the 46.9% average reported by Razieh for the Western Pacific region (9). The substantial disparities in NSI prevalence and underreporting rates among studies underscore the pressing need for a standardized definition of NSI and the establishment of comprehensive reporting systems within China and globally. These measures are essential to ensure accurate data collection and enhance HCW safety worldwide.

In contrast to the prevailing perception held by some healthcare professionals or statistical data from developed states (13, 19), It’s worth noting that even though experienced staff may experience fewer injuries due to improved skills, they may still face increased risks associated with workload and burnout. Our study provides novel evidence that, within the context of the Chinese healthcare system, experienced HCWs (>5-years) display heightened NSIs and underreported rates as opposed to a decline when contrasted with their less experienced counterparts (<1-year).

Our study offers a cautiously approached and unique perspective by dissecting the causes of NSIs and underreported NSIs according to HCWs’ years of service, a dimension that has not been extensively explored in prior research. This novel approach suggests the potential for tailoring HCWs’ training programs to align with their tenure, necessitating careful consideration. Among junior HCWs (<1 year), we have observed a noticeably higher likelihood of injuries attributed to inadequate skills and an increased tendency for non-reporting due to their limited familiarity with established protocols.

Our findings are consistent with prevailing research (18, 22, 33, 34), which suggests that the primary factor contributing to the underreporting of NSI is the perception of minimal infection risk. Unlike studies conducted in Europe and North America (18, 22, 34), our results, akin to Shiao et al.’s (33) investigation in Taiwan, identify a lack of familiarity with reporting procedures as the second major cause of underreporting. Significantly, we observed notable differences in this aspect across various years of work experience. This disparity underscores substantial regional variations in reporting practices, likely influenced by diverse healthcare systems and professional norms. In line with broader research trends, our study emphasizes that a cumbersome reporting process remains a substantial worldwide barrier to the reporting of NSIs. Notably, our findings reveal that this barrier is particularly pronounced in the >1- ≤ 5 years of work experience group. However, contrary to the significant differences in reason for unreported NSI among different tenure groups. There is an absence of statistical differences in the multivariate regression of unreported cases across various tenure groups.

Our research confirms that increasing annual training sessions for NSIs correlates with lower NSI rates. Importantly, training notably influences reporting, with a single session annually increasing reporting by nearly 40%. Comparable effects are also seen in cost reimbursement systems, where institutions and individuals sharing costs boost reporting rates by 23.73%. These results underscore the significant implications of robust training programs for both preventing NSIs and fostering a safety culture within healthcare institutions.

### Limitations

This study’s methodology involves convenience sampling, with participants self-reporting data. This approach inherently introduces a certain level of bias due to the self-reported nature of the data collection. Secondly, the study exclusively includes public medical institutions, omitting private healthcare facilities and individual clinics. Third, a noteworthy limitation arises from the study’s retrospective nature, requiring participants to recall NSIs from the past year. This unavoidably leads to some degree of recall bias. Moreover, due to research constraints in objectives and questionnaire length, the study did not extensively investigate the impact of diverse training durations, types, and effectiveness on NSI incidence and reporting rates. These factors, deemed crucial influencers, necessitate further research to achieve a comprehensive understanding of NSIs among HCWs.

## Conclusion

This cross-sectional study surveyed the prevalence and underreporting rate of NSIs among over 7,000 HCWs in Gansu Province, China. The study population covered physicians, nurses, and other HCWs from public hospitals across all levels and community health centers in the province. The results showed a self-reported NSI incidence of 32.86%, of which 28.53% went unreported. Tertiary hospitals and the surgery department are particularly affected. Experienced healthcare workers, contrary to common belief, exhibit higher NSI and underreporting rates than their less-experienced counterparts. The primary reasons behind NSIs and underreporting are lapses in concentration and not perceiving patients as infectious. However, our findings also emphasize the positive impact of frequent training and improved reimbursement policies on reducing NSIs and encouraging reporting. In light of these results, there is a pressing need for enhanced surveillance, tailored training programs, and more efficient reporting mechanisms to combat this significant occupational health challenge.

## Data availability statement

The raw data supporting the conclusions of this article will be made available by the authors, without undue reservation.

## Ethics statement

The studies involving humans were approved by the Lanzhou University Second Hospital Review Board. The studies were conducted in accordance with the local legislation and institutional requirements. The ethics committee/institutional review board waived the requirement of written informed consent for participation from the participants or the participants’ legal guardians/next of kin because no personal or sensitive information was collected.

## Author contributions

WT: Conceptualization, Formal analysis, Investigation, Methodology, Writing – original draft, Writing – review & editing. LY: Conceptualization, Data curation, Resources, Writing – original draft, Writing – review & editing. WX: Resources, Supervision, Validation, Writing – review & editing. HM: Conceptualization, Data curation, Validation, Writing – review & editing.
