# Supplementary material for: A large-scale survey on epidemiology and underreporting of needlestick and sharp injuries among healthcare workers in China
Source: Front Public Health. 2023 Nov 2;11:1292906. doi: 10.3389/fpubh.2023.1292906 (PMC10652868; doi:10.3389/fpubh.2023.1292906)
Supplement: Supplementary file 1 [file Data_Sheet_1.docx]

**Demographic questions**

1. Your hospital's name and level :
2. Your gender:
3. Your department:
4. Your education level:
5. Your professional title:
6. Your years of service:
7. Your job category:

**Questions regarding occupational exposures and associated factors:**

Occupational Exposure in the Past Year (This survey primarily focuses on occupational exposure related to needlestick and other sharp injuries):

1. Have you experienced occupational exposure in the past year? If yes, please select the reasons.
2. After experiencing occupational exposure in the past year, did you report it?
3. If you did not report occupational exposure in the past year, please select the reasons.
4. Have you received training on the management and reporting of occupational exposure in the past year? If yes, how many times?
5. How were the costs incurred due to occupational exposure in the past year covered?

**The document incorporates seven demographic questions and five items related to occupational exposures and associated factors, translated from the original Chinese questionnaire.**
